# Evaluating the Role of Same-Day Repeat Duplex Ultrasound in Surgical Decision-Making for Carotid Endarterectomy

**DOI:** 10.7759/cureus.105365

**Published:** 2026-03-17

**Authors:** Ahmed Hassan, Ahmed Elshiekh, Alexander Sergiou, Jessica R Helm, Christopher Imray

**Affiliations:** 1 Vascular Surgery, University Hospitals Coventry and Warwickshire NHS Trust, Coventry, GBR

**Keywords:** carotid cancellations, carotid endarterectomy, carotid stenosis, dual antiplatelet therapy, plaque regression, same-day duplex

## Abstract

Background and objective

Carotid artery stenosis is a major cause of ischemic stroke and transient ischemic attack. Timely carotid endarterectomy (CEA) reduces the risk of recurrent stroke in appropriately selected symptomatic patients. However, stenosis severity may change between diagnosis and surgery following initiation of best medical therapy (BMT), potentially influencing operative decision-making. The objective of this study is to evaluate the impact of routine same-day repeat duplex ultrasound as part of a structured preoperative pathway for patients scheduled for CEA, focusing on the detection of plaque regression following BMT and its influence on surgical planning and cancellation.

Methods

This single-center retrospective observational study was performed at University Hospitals Coventry and Warwickshire between January 1, 2019, and June 1, 2021. Duplex ultrasound scans were performed at initial assessment and repeated on the day of surgery. Stenosis severity was classified using institutional reporting standards incorporating peak systolic velocity and velocity ratio criteria. Regression analysis was based on reported percentage stenosis values.

Results

A total of 107 patients were included. Surgery was canceled in 11 patients (10.4%), including two patients (1.9%) in whom same-day duplex ultrasound demonstrated regression of stenosis below 50%. Overall stenosis categorization remained unchanged in most patients; however, 17 patients (15.9%) demonstrated regression greater than 5% between scans. Among the 96 patients who underwent CEA, 30-day outcomes were favorable, with one stroke (1.0%), two hematomas requiring evacuation (2.1%), and no deaths.

Conclusions

Same-day repeat duplex ultrasound changed operative management in a small proportion of patients, with regression sufficient to change indication occurring exclusively in those with moderate (50-69%) baseline stenosis. These findings suggest that routine rescanning may not be necessary, but selective repeat imaging near treatment thresholds may be reasonable.

## Introduction

Carotid artery stenosis is a well-recognized cause of ischemic stroke and represents a significant contributor to global cerebrovascular morbidity and mortality. Carotid endarterectomy (CEA) remains a cornerstone intervention in the management of symptomatic patients, particularly those with high-grade stenosis (>70%), where it has been shown to substantially reduce stroke risk. Importantly, evidence from landmark studies, including the CEA Trialists’ Collaboration, has demonstrated that patients with ≥50% symptomatic stenosis also derive significant benefit from CEA, especially when performed within two weeks of symptom onset [1].

The advent and increasing uptake of best medical therapy (BMT), including dual antiplatelet agents and high-dose statins, have prompted reevaluation of traditional surgical thresholds and management approaches [2]. Recent clinical guidelines recommend reassessment of stenosis severity prior to surgical intervention, particularly in patients receiving optimized medical therapy [3]. However, the existing evidence base remains limited with respect to the dynamic morphological changes in carotid plaque during the preoperative period under BMT.

This retrospective single-center study aims to evaluate the impact of routine same-day repeat duplex ultrasound on surgical decision-making for patients scheduled for CEA, particularly its effect on operative planning and cancellation rates. The secondary objective was to identify plaque regression in carotid stenosis following BMT, defined as a reduction in stenosis severity greater than 5%, including regression resulting in stenosis below the surgical threshold [3,4].

## Materials and methods

Study design and patient selection

This retrospective observational study was performed at University Hospitals Coventry and Warwickshire (UHCW) between January 1, 2019, and June 1, 2021. Patients were included if they had symptomatic ≥50% carotid stenosis, underwent CEA with same-day duplex scanning, and received BMT. Patients were excluded if they had prior ipsilateral CEA, carotid stenting, nonatherosclerotic disease, or incomplete imaging or clinical data.

Following the implementation of a routine same-day repeat duplex ultrasound protocol for patients scheduled for CEA, data were collected retrospectively from electronic patient records. All patients received BMT, including dual antiplatelet agents (aspirin and clopidogrel) and high-dose statins, initiated at the time of presentation with a transient ischemic attack (TIA), ischemic stroke, or related symptoms. TIA and ischemic stroke were defined according to contemporary stroke diagnostic criteria.

The duration of BMT exposure varied depending on the interval between the index cerebrovascular event and the scheduled operation. Accordingly, patients were stratified into two groups: those who underwent CEA within two weeks (“short interval”) and those with an interval of 14 days or more (“long interval”) between scans [5].

Duplex ultrasound protocol

Initial carotid duplex ultrasonography was performed at diagnosis, with repeat scanning on the day of surgery. Each study was independently performed by two different accredited vascular scientists (four independent vascular scientists in total), following a standardized protocol established within the UHCW vascular laboratory to minimize interoperator bias. Stenosis severity was classified according to institutional duplex reporting standards incorporating peak systolic velocity and St Mary’s ratio criteria, consistent with published duplex ultrasound consensus criteria [4].

Regression analysis was based on reported percentage stenosis values extracted from formal reports. No caliper-based or B-mode plaque thickness measurements were included in the regression analyses. Plaque regression was defined as a reduction in stenosis severity greater than 5% between the initial and repeat duplex studies. This threshold was chosen to minimize the impact of expected interobserver and technical variability in duplex ultrasound measurements and to reflect a meaningful interval change rather than measurement noise [6].

Subgroup analysis

In addition to whole-cohort analysis, a subgroup analysis was performed based on the initial degree of stenosis. Patients were categorized into two groups: Group A (initial stenosis 70-99%) and Group B (initial stenosis 50-69%). Within Group B, the proportion of patients whose repeat duplex scan showed regression to <50% stenosis was assessed, along with surgical cancellation status. Fisher’s exact test was used to compare cancellation rates between groups.

Intraoperative monitoring

Intraoperatively, all patients underwent transcranial Doppler (TCD) monitoring, in line with institutional protocols, to guide selective shunting during CEA.

Statistical analysis

Statistical analysis was performed using Python version 3.9 (Python Software Foundation, Wilmington, DE, USA), utilizing the Pandas, SciPy, and Seaborn libraries. Categorical variables are presented as frequencies and percentages, while continuous variables are summarized as medians with interquartile ranges due to nonnormal distribution. Associations between plaque regression and scan interval were assessed using Fisher’s exact test. All statistical tests were two-sided, with a significance threshold of p < 0.05. Data on interoperator variability were not available and are addressed in the limitations section.

Data confidentiality and compliance

All patient data were handled in accordance with the General Data Protection Regulation and the UK National Health Service data protection policies. Study results are reported in an aggregated manner to maintain patient confidentiality.

## Results

A total of 107 patients were included. The study cohort had a mean age of 73.8 ± 9.4 years. The majority were nonsmokers (80%), with 12.5% identified as current smokers and 7.3% as ex-smokers. Hypertension was the most prevalent comorbidity (69.8%), followed by ischemic heart disease (10.4%), chronic obstructive pulmonary disease (8.3%), chronic kidney disease (8.3%), and a history of TIA (6.3%). Less frequent conditions included diabetes mellitus (4.2%), prior stroke (4.2%), depression (4.2%), hyperlipidemia (2.1%), and obstructive sleep apnea (1.0%). Statin therapy had been initiated in 58.3% of patients prior to presentation (Table 1).

**Table 1:** Baseline patient characteristics (n = 107)

Characteristic	n	%
Nonsmoker	86	80
Current smoker	13	12.5
Ex-smoker	8	7.3
Hypertension	75	69.8
Ischemic heart disease	11	10.4
Chronic obstructive pulmonary disease	9	8.3
Chronic kidney disease	9	8.3
History of transient ischemic attack	7	6.3
Diabetes mellitus	4	4.2
Prior stroke	4	4.2
Depression	4	4.2
Hyperlipidemia	2	2.1
Obstructive sleep apnea	1	1.0
Preexisting statin therapy	62	58.3

**Figure 1:**
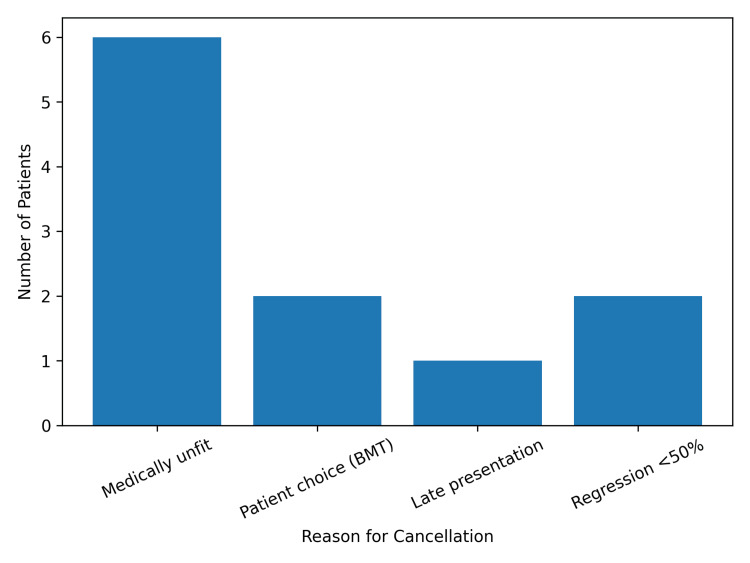
Reasons for CEA cancellation (n = 11) BMT, best medical therapy; CEA, carotid endarterectomy

CEA was canceled in 11 patients (10.4%) following repeat duplex ultrasound. The reasons for cancellation are illustrated in Figure 1.

Of these, six patients (5.7%) were deemed medically unfit for surgery by a vascular anesthetist. Two additional patients (1.9%) opted to continue with BMT alone, and one patient (0.9%) presented beyond three months from symptom onset, resulting in deferral due to unfavorable risk-benefit considerations. In two patients (1.9%), surgery was canceled because same-day repeat duplex ultrasound demonstrated regression of stenosis to below the 50% threshold (Figure 1).

**Figure 2:**
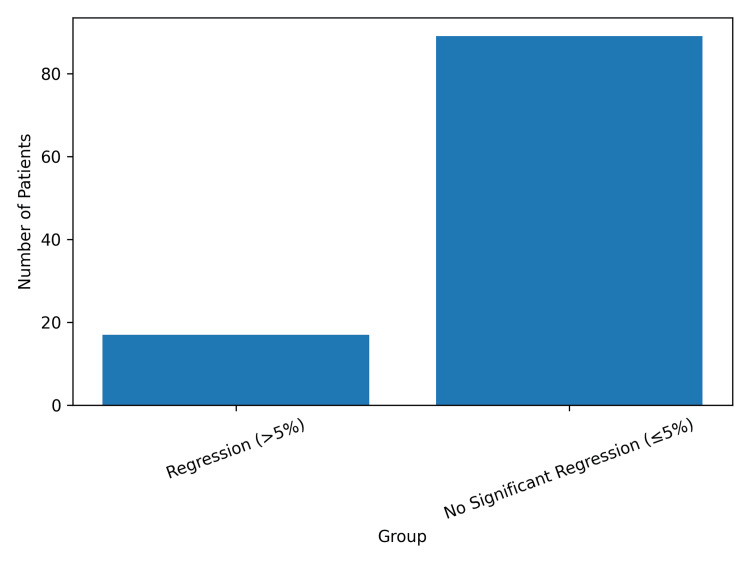
Change in reported percentage stenosis by regression status

Repeat duplex ultrasound demonstrated changes in the reported degree of carotid stenosis in a subset of patients. Changes in reported percentage stenosis following same-day repeat duplex ultrasound varied according to regression status, as shown in Figure 2.

**Figure 3:**
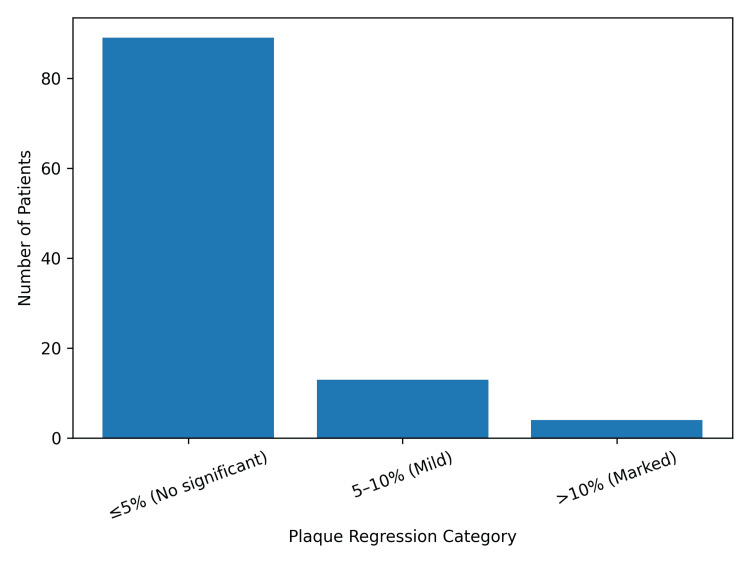
Distribution of plaque regression across the study cohort (n = 107) Proportion of patients demonstrating ≤5% change, 5-10% regression, and >10% regression in stenosis severity between initial and repeat duplex ultrasound examinations.

No change in stenosis categorization (≤5% regression) was observed in 89 patients (83.2%). Regression greater than 5% between scans was identified in 17 patients (15.9%). The distribution of plaque regression across the study cohort is shown in Figure 3.

**Figure 4:**
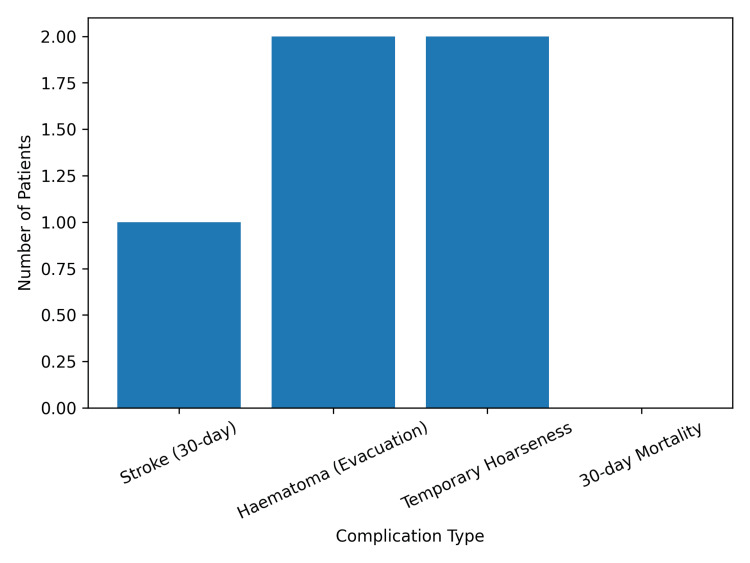
Post-CEA complication rates CEA, carotid endarterectomy

Postoperative complications were infrequent. A 30-day stroke occurred in one patient (1.0%), temporary hoarseness of voice occurred in two patients (2.08%), and postoperative hematomas requiring surgical drainage were reported in two patients (2.08%). Myocardial infarction was documented in two patients (2.08%) within the first year; however, no myocardial events occurred within the first 30 days. There were no deaths reported within the 30-day postoperative period. These findings are summarized in Figure 4.

Plaque regression was observed in 17 patients (15.9%), with an average reduction of 12.3%. Among the two patients whose stenosis regressed to <50%, one underwent repeat scanning in week 1 and the other in week 3 following symptom onset.

Subgroup analysis showed that of the 31 patients with initial stenosis between 50% and 69% (Group B), two were canceled because stenosis regressed to below 50% on same-day repeat duplex ultrasound. In contrast, among the 76 patients with initial stenosis of 70-99% (Group A), no cancellations occurred due to regression, and no patients progressed to complete occlusion. The difference in regression-related cancellation between groups did not reach statistical significance (Fisher’s exact test, p = 0.082).

## Discussion

The observed reduction in carotid stenosis among patients showing plaque regression reinforces the potential therapeutic efficacy of BMT in the context of symptomatic carotid artery stenosis. These findings highlight the potential value of same-day repeat duplex ultrasound in guiding surgical decision-making, offering a strategy to avoid unnecessary operative intervention in selected cases. The low 30-day stroke rate observed in our cohort is consistent with recent reports of low perioperative neurologic event rates after CEA [7].

Significant plaque regression was identified within a median interval of approximately 10 days following initiation of dual antiplatelet therapy and high-intensity statin treatment. Greater regression was observed in patients with longer exposure to BMT. This suggests a time-dependent response, potentially attributable in part to the antithrombotic effects of dual antiplatelet therapy. However, while dual antiplatelet therapy may influence thrombotic burden and plaque stabilization, perioperative bleeding risks must also be considered when managing patients undergoing carotid intervention [8,9].

These findings should be interpreted with caution, given the potential influence of measurement variability and the relatively short follow-up interval. It is essential to note that the possibility of medical therapy-induced regression should not delay surgical intervention in symptomatic patients, particularly within the critical 14-day window, as current guidelines continue to recommend timely CEA to minimize recurrent stroke risk [1,10].

Previous MRI-based studies of statin therapy have demonstrated plaque composition changes over months rather than weeks, suggesting that early duplex changes may reflect hemodynamic or thrombotic modulation rather than structural remodeling [11,12].

Routine use of intraoperative TCD monitoring to guide selective shunting contributed to operative safety across all cases at our institution. The integration of routine preoperative repeat duplex scanning, particularly in patients undergoing BMT, may help refine surgical indication and potentially exclude individuals whose stenosis has regressed below operative thresholds [13].

Although plaque regression was observed in 17 patients (15.9% of the cohort), this anatomical improvement led to a change in surgical indication in only two patients (1.9%). Notably, both cases occurred in patients with moderate baseline stenosis (50-69%), suggesting that same-day repeat duplex scanning is particularly valuable in this subgroup, where regression to below the 50% threshold can alter management. In contrast, no such cancellations occurred among patients with higher-grade stenosis (70-99%), and no cases progressed to complete occlusion, indicating the limited clinical value of repeat imaging in this group, particularly with the use of BMT. These findings highlight the importance of targeted use of repeat imaging in patients most likely to benefit.

Furthermore, the modest impact on surgical decision-making may also reflect the relatively short interval between scans, which may be insufficient to allow substantial plaque remodeling. While medical therapy can promote regression, more prolonged exposure may be required to produce clinically significant changes.

Several limitations should be acknowledged. As a retrospective, single-center analysis, the findings may be subject to selection bias and may not be generalizable to other institutions. Adherence to BMT was presumed based on prescription records, not direct measurement. Importantly, despite a standardized imaging protocol, the inherent operator dependency of duplex ultrasound may have influenced the measurement of stenosis severity. Some of the plaque regression or progression identified may reflect interoperator variability rather than true biological change. Future studies incorporating additional imaging modalities or independent adjudication of scans could help mitigate this bias. Minor changes in measured stenosis, particularly those within ±5%, may reflect interoperator variability rather than true biological regression.

Raw Doppler velocity measurements were not available for retrospective verification, and percentage stenosis values were extracted from formal reports. Therefore, subtle changes in velocity parameters could not be independently analyzed.

## Conclusions

Same-day repeat duplex ultrasound resulted in a change in operative management in a small proportion of patients in this cohort. BMT was associated with short-term reductions in duplex-estimated stenosis severity in some individuals; however, regression sufficient to alter surgical indication occurred in only 1.9% of cases. These findings suggest that routine rescanning of all patients awaiting CEA may not be justified. All cancellations due to stenosis regression occurred in patients with moderate (50-69%) baseline stenosis, while repeat imaging did not affect management in those with high-grade (70-99%) disease. This pattern supports a selective, threshold-based approach to repeat duplex assessment, particularly in patients whose stenosis lies close to the 50% threshold. Larger prospective studies are needed to determine whether targeted repeat imaging improves clinical outcomes and optimizes resource utilization.
